# Synthesis and crystal structure of (*E*)-2-({2-[aza­niumyl­idene(methyl­sulfan­yl)meth­yl]hydrazinyl­idene}meth­yl)benzene-1,4-diol hydrogen sulfate

**DOI:** 10.1107/S2056989019014233

**Published:** 2019-10-29

**Authors:** Oussama Nehar, Samira Louhibi, Thierry Roisnel

**Affiliations:** a Laboratoire de Chimie Inorganique et Environnement, Université de Tlemcen, BP 119, 13000 Tlemcen, Algeria; b Centre de Diffractometrie X, UMR 6226 CNRS, Unit Sciences Chimiques de Rennes, Universite de Rennes I, 263 Avenue du General Leclerc, 35042 Rennes, France

**Keywords:** crystal structure, thio­semicarbazone, hydrogen bonding, organic salt

## Abstract

The title mol­ecular salt, C_9_H_12_N_3_O_2_S^+^·HSO_4_^−^, was obtained through the protonation of the azomethine N atom in a sulfuric acid medium. The crystal com­prises two entities, a thio­semicarbazide cation and a hydrogen sulfate anion. The cation is essentially planar and is further stabilized by a strong intra­molecular O—H⋯N hydrogen bond. In the crystal, a three-dimensional network is established through O—H⋯O and N—H⋯O hydrogen bonds. A weak intermolecular C—H⋯O hydrogen bond is also observed. The hydrogen sulfate anion exhibits disorder over two sets of sites and was modelled with refined occupancies of 0.501 (6) and 0.499 (6).

## Abstract 2

The title mol­ecular salt was obtained through the protonation of the azomethine N atom in a sulfuric acid medium. The crystal com­prises two entities, a thio­semicarbazide cation and a hydrogen sulfate anion. The cation is essentially planar and is further stabilized by a strong intra­molecular O—H⋯N hydrogen bond.

## Chemical context   

Thio­semicarbazones and their com­plexes are well known for their pharmacological properties, as anti­microbial **(Plech *et al.*, 2011 ▸;** Pandeya *et al.*, 1999 ▸; Küçükgüzel *et al.*, 2006 ▸), anti-inflammatory (Palaska *et al.*, 2002 ▸) and anti­umoural (de Oliveira *et al.*, 2015 ▸) agents. Complexes of thio­semicarbazones are studied in the literature as drug candidates, biomarkers and biocatalysts (Hayne *et al.*, 2014 ▸; Lim *et al.*, 2010 ▸). It is believed that the biological activity of these com­pounds has a strong relationship with the nature of the aldehydes and ketones from which those thio­semicarbazones were obtained (Teoh *et al.*, 1999 ▸), and also on the substituents attached at the ^+^NH_2_ N atom (Beraldo & Gambino, 2004 ▸). An inter­esting attribute of thio­semicarbazones is their ability to exhibit thione–thiol tautomerism and they can also exist as *E* and *Z* isomers. Thio­semicarbazones have an excellent capacity to com­plex transition metals, acting as chelating agents; this process usually takes place *via* dissociation of the acidic proton (Pal *et al.*, 2002 ▸). The crystal structure of the title mol­ecular salt was determined in order to investigate its biological and catalytic activities.

## Structural commentary   

**Figure 1:**
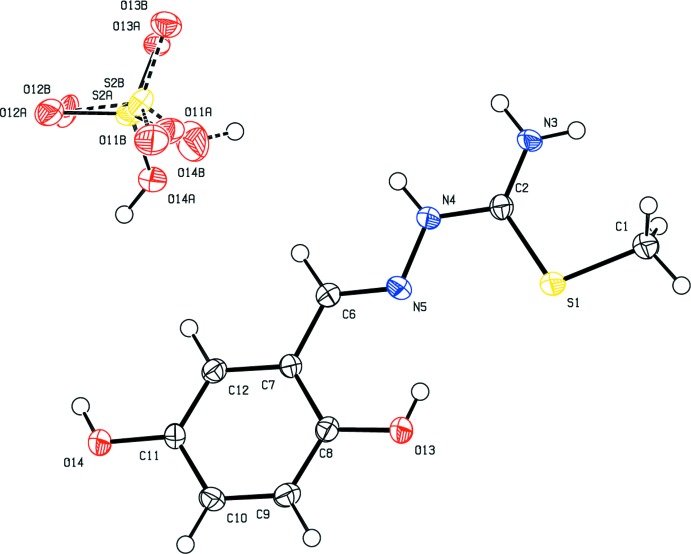
The mol­ecular structure of the title mol­ecular salt, showing the labelling and with displacement ellipsoids drawn at the 50% probability level. The disordered hydrogen sulfate anion is shown.

The mol­ecular structure of the title mol­ecular salt is illustrated in Fig. 1 ▸. It com­prises two entities, *i.e.* a thio­semicarbazone cation and a hydrogen sulfate anion. The cation is essentially planar and shows an *E* conformation with regard to the C6—N5 bond, the maximum deviation from the mean plane through the 15 non-H atoms being 0.1 (2) Å for atom C6. This planarity is due to electron delocalization along the cation backbone, which is further stabilized by an intra­molecular O13—H13⋯N5 hydrogen bond (Zhu *et al.*, 2004 ▸). The bond lengths and angles resemble those observed for similar thio­semicarbazone derivatives (Gangadharan *et al.*, 2015 ▸; Joseph *et al.*, 2004 ▸; Nehar *et al.*, 2016 ▸; Houari *et al.*, 2013 ▸). The anion (hydrogen sulfate) is disordered, split over two sets of siteswith relative occupancies of 0.501 (6) and 0.499 (6), and labelled with *A* and *B* suffixes.
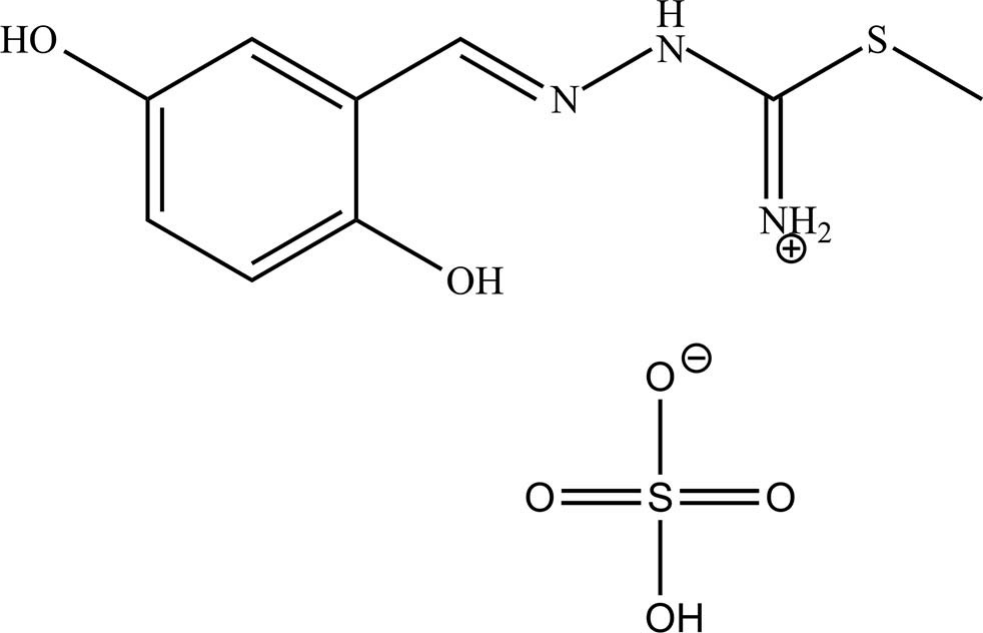


## Supra­molecular features   

**Figure 2:**
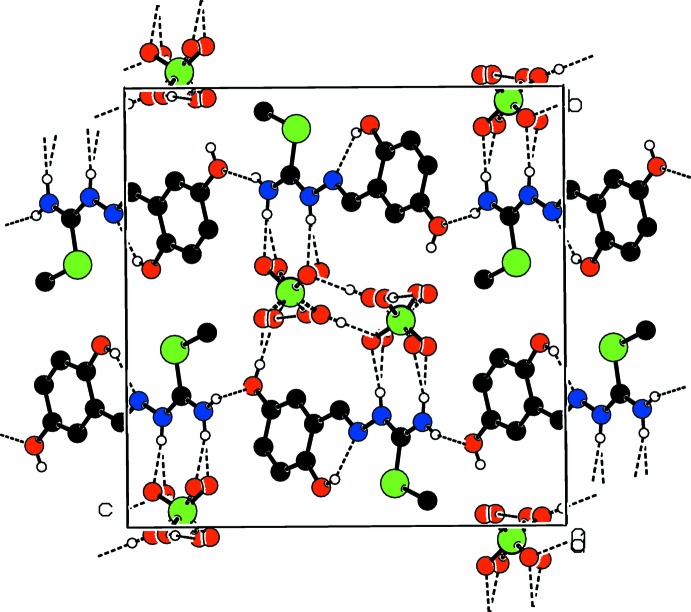
Projection along the *a* axis of the crystal packing of the title mol­ecular salt. Hydrogen bonds are shown as dashed lines.

In the crystal, the three-dimensional structure is established through an extensive network of O—H⋯O and N—H⋯O hydrogen bonds. Also within this network exists a weak C—H⋯O inter­molecular hydrogen bond (Table 1 ▸ and Fig. 2 ▸). The crystal packing is shown in Fig. 2 ▸.

## Database survey   

A search in the Cambridge Structural Database (CSD, Version 5.4, May 2019 update; Groom *et al.*, 2016 ▸) for the *S*-meth­yl(methyl­idene)thio­semicarbazidium cation yielded three results, *viz. S*-methyl-*N*-(pyrrolyl-2-methyl­ene)iso­thio­semi­car­bazidium iodide monohydrate (CSD refcode JIHZUV; Bourosh *et al.*, 1990 ▸), 8-quinoline­aldehyde *S*-methyl­thio­semicarbazone hydro­chloride dihydrate (RUJXOK; Botoshansky *et al.*, 2009 ▸) and ((*E*)-{2-[(*E*)-(4-hy­droxy­naphthalen-1-yl)methyl­idene]hydrazin-1-yl}(methyl­sulfan­yl)methyl­idene)aza­nium hydrogen sulfate monohydrate. The three-dimensional coordinates for the first structure are unavailable. A com­parison of the structures reveals that the cation in the RUJXOK structure is less planar than the cation in ESOTIR, the latter being more similar to the cation of the title com­pound. However, for structures RUJXOK and ESOTIR, the bond lengths and angles are similar to those of the title mol­ecular salt.

## Synthesis and crystallization   

An equimolar amount of thio­semicarbazide (10 mmol, 0.91 g) and 2,5-di­hydroxy­benzaldehyde (10 mmol, 1.38 g) were dissolved in a methanol–water solution in the presence of sulfuric acid. The mixture was then refluxed for 3 h. The solution was filtered and left to evaporate at room temperature. After slow evaporation, brown crystals suitable for X-ray diffraction analysis were obtained.

## Refinement   

Crystal data, data collection and structure refinement details are summarized in Table 2 ▸. The hydrogen sulfate anion is disordered and had to be modelled as two conformations *A* and *B*, with relative occupancies of 0.501 (6) and 0.499 (6), respectively. H atoms were located in difference Fourier maps, but were subsequently included in calculated positions and treated as riding on their parent atoms with constrained thermal parameters: *U*_iso_(H) = 1.5*U*_eq_(C) and C—H = 0.98 Å for methyl H atoms, and *U*_iso_(H) = 1.2*U*_eq_(C,N) and C—H = 0.95 Å or N—H = 0.88 Å otherwise.

## Supplementary Material

Crystal structure: contains datablock(s) global. DOI: 10.1107/S2056989019014233/lh5926sup1.cif

CCDC references: 1960006, 1960006

Additional supporting information:  crystallographic information; 3D view; checkCIF report

## Figures and Tables

**Table 1:** Hydrogen-bond geometry (Å, °)

*D*—H⋯*A*	*D*—H	H⋯*A*	*D*⋯*A*	*D*—H⋯*A*
C10—H10⋯O12*A* ^i^	0.95	2.60	3.541 (10)	170
N3—H3*A*⋯O12*A* ^ii^	0.89 (2)	1.85 (2)	2.738 (12)	178 (3)
N3—H3*A*⋯O12*B* ^ii^	0.89 (2)	1.98 (2)	2.841 (11)	163 (3)
N3—H3*B*⋯O14^iii^	0.86 (2)	2.05 (2)	2.874 (3)	160 (3)
N4—H4⋯O13*A* ^ii^	0.86 (3)	2.00 (3)	2.849 (5)	167 (3)
N4—H4⋯O13*B* ^ii^	0.86 (3)	2.00 (3)	2.841 (5)	164 (3)
O13—H13⋯N5	0.78 (3)	2.03 (3)	2.685 (3)	142 (3)
O14—H14⋯O11*A* ^iv^	0.83 (4)	1.90 (4)	2.716 (16)	167 (3)
O14—H14⋯O11*B* ^iv^	0.83 (4)	1.82 (4)	2.62 (2)	162 (3)
O14*A*—H14*A*⋯O11*A* ^iv^	0.84	2.28	3.123 (17)	180
O14*B*—H14*B*⋯S2*B* ^ii^	0.84	2.73	3.490 (9)	152
O14*B*—H14*B*⋯O13*B* ^ii^	0.84	1.73	2.567 (7)	180

Symmetry codes: (i) 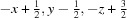; (ii) 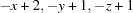; (iii) 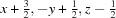; (iv) 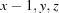.

**Table 2:** Experimental details

Crystal data	
Chemical formula	C_9_H_12_N_3_O_2_S^+^·HSO_4_^−^
*M*_r_	323.34
Crystal system, space group	Monoclinic, *P*2_1_/*n*
Temperature (K)	150
*a*, *b*, *c* (Å)	4.9411 (8), 16.139 (2), 16.426 (3)
β (°)	100.440 (7)
*V* (Å^3^)	1288.2 (3)
*Z*	4
Radiation type	Mo *K*α
μ (mm^−1^)	0.44
Crystal size (mm)	0.38 × 0.15 × 0.12

Data collection	
Diffractometer	Bruker APEXII
Absorption correction	Multi-scan (*SADABS*; Bruker, 2015 ▸)
*T*_min_, *T*_max_	0.838, 0.948
No. of measured, independent and observed [*I* > 2σ(*I*)] reflections	7962, 2846, 2014
*R*_int_	0.045
(sin θ/λ)_max_ (Å^−1^)	0.644

Refinement	
*R*[*F* ^2^ > 2σ(*F* ^2^)], *wR*(*F* ^2^), *S*	0.050, 0.147, 1.03
No. of reflections	2846
No. of parameters	243
No. of restraints	8
H-atom treatment	H atoms treated by a mixture of independent and constrained refinement
Δρ_max_, Δρ_min_ (e Å^−3^)	0.36, −0.46

Computer programs: *APEX2* (Bruker, 2015 ▸), *SAINT* (Bruker, 2015 ▸), *SHELXT* (Sheldrick, 2015*a* ▸), *SHELXL2018* (Sheldrick, 2015*b* ▸), *SXGRAPH* (Farrugia, 1999 ▸), *Mercury* (Macrae *et al.*, 2008 ▸) and *CRYSCALC* (T. Roisnel, local program).

## supplementary crystallographic information

### Crystal data

**Table:**

C_9_H_12_N_3_O_2_S^+^·HSO_4_^−^	*F*(000) = 672
*M**_r_* = 323.34	*D*_x_ = 1.667 Mg m^−^^3^
Monoclinic, *P*2_1_/*n*	Mo *K*α radiation, λ = 0.71073 Å
*a* = 4.9411 (8) Å	Cell parameters from 2859 reflections
*b* = 16.139 (2) Å	θ = 2.5–27.0°
*c* = 16.426 (3) Å	µ = 0.44 mm^−^^1^
β = 100.440 (7)°	*T* = 150 K
*V* = 1288.2 (3) Å^3^	Prism, colourless
*Z* = 4	0.38 × 0.15 × 0.12 mm

### Data collection

**Table:**

Bruker APEXII diffractometer	2846 independent reflections
Radiation source: fine-focus sealed tube	2014 reflections with *I* > 2σ(*I*)
Graphite monochromator	*R*_int_ = 0.045
CCD rotation images, thin slices scans	θ_max_ = 27.3°, θ_min_ = 3.6°
Absorption correction: multi-scan (SADABS; Sheldrick, 2014)	*h* = −6→4
*T*_min_ = 0.838, *T*_max_ = 0.948	*k* = −19→20
7962 measured reflections	*l* = −21→19

### Refinement

**Table:**

Refinement on *F*^2^	Primary atom site location: dual
Least-squares matrix: full	Secondary atom site location: difference Fourier map
*R*[*F*^2^ > 2σ(*F*^2^)] = 0.050	Hydrogen site location: mixed
*wR*(*F*^2^) = 0.147	H atoms treated by a mixture of independent and constrained refinement
*S* = 1.03	*w* = 1/[σ^2^(*F*_o_^2^) + (0.086*P*)^2^] where *P* = (*F*_o_^2^ + 2*F*_c_^2^)/3
2846 reflections	(Δ/σ)_max_ < 0.001
243 parameters	Δρ_max_ = 0.36 e Å^−^^3^
8 restraints	Δρ_min_ = −0.46 e Å^−^^3^

### Special details

**Table:**

Geometry. All esds (except the esd in the dihedral angle between two l.s. planes) are estimated using the full covariance matrix. The cell esds are taken into account individually in the estimation of esds in distances, angles and torsion angles; correlations between esds in cell parameters are only used when they are defined by crystal symmetry. An approximate (isotropic) treatment of cell esds is used for estimating esds involving l.s. planes.
Refinement. Refinement of F^2^ against ALL reflections. The weighted R-factor wR and goodness of fit S are based on F^2^, conventional R-factors R are based on F, with F set to zero for negative F^2^. The threshold expression of F^2^ > 2sigma(F^2^) is used only for calculating R-factors(gt) etc. and is not relevant to the choice of reflections for refinement. R-factors based on F^2^ are statistically about twice as large as those based on F, and R- factors based on ALL data will be even larger.

### Fractional atomic coordinates and isotropic or equivalent isotropic displacement parameters (Å^2^)

**Table:**

	*x*	*y*	*z*	*U*_iso_*/*U*_eq_	Occ. (<1)
S1	0.99180 (14)	0.09724 (4)	0.39012 (4)	0.0247 (2)	
C1	1.1843 (6)	0.05832 (18)	0.31564 (19)	0.0283 (7)	
H1A	1.103765	0.079127	0.260512	0.042*	
H1B	1.178644	−0.002377	0.315474	0.042*	
H1C	1.375761	0.076956	0.330229	0.042*	
C2	1.0137 (5)	0.20289 (18)	0.37549 (16)	0.0196 (6)	
N3	1.1584 (4)	0.23722 (15)	0.32555 (15)	0.0212 (5)	
H3A	1.163 (6)	0.2922 (11)	0.3217 (18)	0.025*	
H3B	1.253 (5)	0.2090 (17)	0.2961 (16)	0.025*	
N4	0.8734 (5)	0.25101 (15)	0.41903 (15)	0.0221 (5)	
H4	0.897 (6)	0.304 (2)	0.4220 (19)	0.027*	
N5	0.7288 (4)	0.21422 (15)	0.47329 (14)	0.0209 (5)	
C6	0.6012 (5)	0.26348 (17)	0.51479 (17)	0.0207 (6)	
H6	0.611234	0.321565	0.506437	0.025*	
C7	0.4421 (5)	0.23214 (17)	0.57410 (16)	0.0179 (6)	
C8	0.4293 (5)	0.14784 (17)	0.59347 (17)	0.0204 (6)	
C9	0.2738 (5)	0.12280 (18)	0.65151 (18)	0.0239 (6)	
H9	0.265269	0.065663	0.664710	0.029*	
C10	0.1324 (5)	0.17925 (17)	0.69007 (17)	0.0225 (6)	
H10	0.025988	0.161037	0.729373	0.027*	
C11	0.1446 (5)	0.26363 (17)	0.67161 (17)	0.0199 (6)	
C12	0.2983 (5)	0.28879 (17)	0.61429 (16)	0.0209 (6)	
H12	0.306923	0.346085	0.601728	0.025*	
O13	0.5640 (4)	0.08764 (12)	0.55849 (13)	0.0270 (5)	
H13	0.648 (7)	0.106 (2)	0.527 (2)	0.032*	
O14	0.0054 (4)	0.31741 (13)	0.71335 (13)	0.0279 (5)	
H14	0.001 (6)	0.366 (2)	0.697 (2)	0.034*	
S2A	0.8844 (12)	0.5353 (4)	0.6254 (4)	0.0254 (10)	0.501 (6)
O11A	1.081 (3)	0.4743 (10)	0.6620 (9)	0.031 (3)	0.501 (6)
O12A	0.810 (2)	0.5938 (7)	0.6848 (5)	0.030 (2)	0.501 (6)
O13A	0.9688 (11)	0.5797 (2)	0.5572 (3)	0.0297 (14)	0.501 (6)
O14A	0.6216 (9)	0.4839 (3)	0.5891 (3)	0.0302 (13)	0.501 (6)
H14A	0.476218	0.481337	0.608697	0.045*	0.501 (6)
S2B	0.9553 (12)	0.5327 (5)	0.6250 (5)	0.0292 (12)	0.499 (6)
O11B	1.084 (4)	0.4751 (12)	0.6870 (8)	0.038 (3)	0.499 (6)
O12B	0.767 (2)	0.5891 (7)	0.6534 (6)	0.038 (2)	0.499 (6)
O13B	1.1639 (10)	0.5733 (3)	0.5857 (3)	0.0294 (13)	0.499 (6)
O14B	0.7723 (10)	0.4819 (4)	0.5562 (3)	0.0506 (17)	0.499 (6)
H14B	0.793868	0.463761	0.509852	0.076*	0.499 (6)

### Atomic displacement parameters (Å^2^)

**Table:**

	*U*^11^	*U*^22^	*U*^33^	*U*^12^	*U*^13^	*U*^23^
S1	0.0307 (4)	0.0178 (4)	0.0285 (4)	−0.0005 (3)	0.0136 (3)	0.0018 (3)
C1	0.0313 (14)	0.0220 (15)	0.0354 (18)	0.0012 (12)	0.0162 (13)	0.0003 (13)
C2	0.0205 (11)	0.0207 (14)	0.0172 (14)	−0.0001 (11)	0.0027 (11)	−0.0014 (11)
N3	0.0252 (11)	0.0175 (12)	0.0236 (13)	0.0011 (10)	0.0114 (10)	0.0049 (10)
N4	0.0285 (11)	0.0178 (12)	0.0228 (13)	−0.0013 (10)	0.0121 (10)	0.0013 (10)
N5	0.0226 (10)	0.0229 (12)	0.0188 (12)	−0.0018 (9)	0.0079 (9)	0.0010 (10)
C6	0.0230 (11)	0.0178 (14)	0.0220 (15)	−0.0004 (11)	0.0057 (11)	0.0001 (11)
C7	0.0197 (11)	0.0182 (13)	0.0162 (14)	−0.0007 (10)	0.0040 (10)	−0.0011 (11)
C8	0.0214 (11)	0.0185 (14)	0.0211 (15)	−0.0003 (10)	0.0035 (11)	−0.0024 (11)
C9	0.0285 (13)	0.0185 (14)	0.0252 (15)	−0.0032 (12)	0.0063 (12)	0.0006 (12)
C10	0.0249 (12)	0.0238 (15)	0.0201 (15)	−0.0030 (11)	0.0075 (11)	0.0009 (12)
C11	0.0214 (11)	0.0191 (14)	0.0202 (14)	0.0002 (11)	0.0064 (11)	−0.0040 (11)
C12	0.0244 (12)	0.0172 (14)	0.0213 (15)	−0.0008 (11)	0.0043 (11)	0.0002 (11)
O13	0.0342 (11)	0.0182 (10)	0.0329 (13)	0.0024 (9)	0.0175 (9)	−0.0009 (9)
O14	0.0363 (10)	0.0186 (10)	0.0336 (12)	0.0014 (9)	0.0186 (9)	−0.0015 (9)
S2A	0.038 (3)	0.0164 (14)	0.0231 (13)	−0.0028 (16)	0.0076 (16)	0.0061 (9)
O11A	0.028 (3)	0.020 (3)	0.043 (8)	0.003 (3)	0.007 (5)	0.010 (5)
O12A	0.040 (4)	0.026 (3)	0.026 (4)	−0.010 (3)	0.009 (3)	−0.005 (3)
O13A	0.050 (3)	0.018 (2)	0.026 (3)	−0.004 (2)	0.021 (2)	0.0035 (18)
O14A	0.031 (2)	0.025 (2)	0.038 (3)	−0.0049 (18)	0.016 (2)	−0.0021 (19)
S2B	0.037 (3)	0.0182 (14)	0.0338 (15)	−0.0054 (16)	0.0117 (17)	−0.0075 (10)
O11B	0.051 (4)	0.030 (4)	0.035 (7)	−0.001 (3)	0.014 (5)	0.007 (5)
O12B	0.038 (4)	0.017 (3)	0.062 (7)	0.005 (3)	0.021 (5)	−0.011 (5)
O13B	0.030 (3)	0.025 (2)	0.036 (3)	0.0028 (19)	0.014 (2)	0.008 (2)
O14B	0.042 (3)	0.068 (4)	0.044 (3)	−0.018 (3)	0.012 (3)	−0.029 (3)

### Geometric parameters (Å, º)

**Table:**

S1—C2	1.728 (3)	C9—H9	0.9500
S1—C1	1.794 (3)	C10—C11	1.399 (4)
C1—H1A	0.9800	C10—H10	0.9500
C1—H1B	0.9800	C11—O14	1.367 (3)
C1—H1C	0.9800	C11—C12	1.374 (3)
C2—N3	1.305 (3)	C12—H12	0.9500
C2—N4	1.332 (3)	O13—H13	0.78 (3)
N3—H3A	0.890 (18)	O14—H14	0.83 (4)
N3—H3B	0.861 (17)	S2A—O11A	1.435 (8)
N4—N5	1.374 (3)	S2A—O12A	1.452 (7)
N4—H4	0.86 (3)	S2A—O13A	1.453 (6)
N5—C6	1.285 (3)	S2A—O14A	1.564 (8)
C6—C7	1.449 (3)	O14A—H14A	0.8399
C6—H6	0.9500	S2B—O12B	1.437 (7)
C7—C12	1.395 (4)	S2B—O11B	1.439 (8)
C7—C8	1.401 (4)	S2B—O13B	1.467 (6)
C8—O13	1.362 (3)	S2B—O14B	1.549 (9)
C8—C9	1.388 (4)	O14B—H14B	0.8401
C9—C10	1.371 (4)		
			
C2—S1—C1	101.32 (13)	C10—C9—H9	119.5
S1—C1—H1A	109.5	C8—C9—H9	119.5
S1—C1—H1B	109.5	C9—C10—C11	120.1 (2)
H1A—C1—H1B	109.5	C9—C10—H10	120.0
S1—C1—H1C	109.5	C11—C10—H10	120.0
H1A—C1—H1C	109.5	O14—C11—C12	123.2 (3)
H1B—C1—H1C	109.5	O14—C11—C10	117.6 (2)
N3—C2—N4	119.2 (3)	C12—C11—C10	119.2 (2)
N3—C2—S1	124.2 (2)	C11—C12—C7	121.5 (3)
N4—C2—S1	116.7 (2)	C11—C12—H12	119.2
C2—N3—H3A	119.5 (19)	C7—C12—H12	119.2
C2—N3—H3B	123 (2)	C8—O13—H13	112 (2)
H3A—N3—H3B	118 (3)	C11—O14—H14	115 (2)
C2—N4—N5	118.6 (2)	O11A—S2A—O12A	113.5 (9)
C2—N4—H4	122 (2)	O11A—S2A—O13A	113.2 (7)
N5—N4—H4	118 (2)	O12A—S2A—O13A	109.8 (7)
C6—N5—N4	116.1 (2)	O11A—S2A—O14A	104.4 (10)
N5—C6—C7	121.3 (3)	O12A—S2A—O14A	107.9 (5)
N5—C6—H6	119.4	O13A—S2A—O14A	107.6 (5)
C7—C6—H6	119.4	S2A—O14A—H14A	126.1
C12—C7—C8	118.7 (2)	O12B—S2B—O11B	114.1 (9)
C12—C7—C6	118.3 (2)	O12B—S2B—O13B	114.0 (7)
C8—C7—C6	123.0 (2)	O11B—S2B—O13B	110.1 (9)
O13—C8—C9	117.1 (2)	O12B—S2B—O14B	104.2 (6)
O13—C8—C7	123.4 (2)	O11B—S2B—O14B	107.4 (11)
C9—C8—C7	119.5 (2)	O13B—S2B—O14B	106.2 (5)
C10—C9—C8	121.0 (3)	S2B—O14B—H14B	133.8
			
C1—S1—C2—N3	−4.6 (3)	C6—C7—C8—C9	179.5 (2)
C1—S1—C2—N4	175.8 (2)	O13—C8—C9—C10	179.7 (2)
N3—C2—N4—N5	−178.0 (2)	C7—C8—C9—C10	0.1 (4)
S1—C2—N4—N5	1.6 (3)	C8—C9—C10—C11	−0.4 (4)
C2—N4—N5—C6	178.8 (2)	C9—C10—C11—O14	−178.4 (2)
N4—N5—C6—C7	−179.8 (2)	C9—C10—C11—C12	0.3 (4)
N5—C6—C7—C12	−177.1 (2)	O14—C11—C12—C7	178.7 (2)
N5—C6—C7—C8	3.7 (4)	C10—C11—C12—C7	0.1 (4)
C12—C7—C8—O13	−179.3 (2)	C8—C7—C12—C11	−0.4 (4)
C6—C7—C8—O13	−0.1 (4)	C6—C7—C12—C11	−179.6 (2)
C12—C7—C8—C9	0.3 (4)		

### Hydrogen-bond geometry (Å, º)

**Table:**

*D*—H···*A*	*D*—H	H···*A*	*D*···*A*	*D*—H···*A*
C10—H10···O12*A*^i^	0.95	2.60	3.541 (10)	170
N3—H3*A*···O12*A*^ii^	0.89 (2)	1.85 (2)	2.738 (12)	178 (3)
N3—H3*A*···O12*B*^ii^	0.89 (2)	1.98 (2)	2.841 (11)	163 (3)
N3—H3*B*···O14^iii^	0.86 (2)	2.05 (2)	2.874 (3)	160 (3)
N4—H4···O13*A*^ii^	0.86 (3)	2.00 (3)	2.849 (5)	167 (3)
N4—H4···O13*B*^ii^	0.86 (3)	2.00 (3)	2.841 (5)	164 (3)
O13—H13···N5	0.78 (3)	2.03 (3)	2.685 (3)	142 (3)
O14—H14···O11*A*^iv^	0.83 (4)	1.90 (4)	2.716 (16)	167 (3)
O14—H14···O11*B*^iv^	0.83 (4)	1.82 (4)	2.62 (2)	162 (3)
O14*A*—H14*A*···O11*A*^iv^	0.84	2.28	3.123 (17)	180
O14*B*—H14*B*···S2*B*^ii^	0.84	2.73	3.490 (9)	152
O14*B*—H14*B*···O13*B*^ii^	0.84	1.73	2.567 (7)	180

Symmetry codes: (i) −*x*+1/2, *y*−1/2, −*z*+3/2; (ii) −*x*+2, −*y*+1, −*z*+1; (iii) *x*+3/2, −*y*+1/2, *z*−1/2; (iv) *x*−1, *y*, *z*.
